# Technology and Electrophysical Properties of PZT-Type Ceramics Doped by Samarium

**DOI:** 10.3390/ma18081773

**Published:** 2025-04-13

**Authors:** Dariusz Bochenek, Dagmara Brzezińska, Przemysław Niemiec, Maciej Zubko, Katarzyna Osińska

**Affiliations:** 1 Institute of Materials Engineering, Faculty of Science and Technology, University of Silesia in Katowice, 75 Pułku Piechoty 1a, 41-500 Chorzów, Poland; dariusz.bochenek@us.edu.pl (D.B.); maciej.zubko@us.edu.pl (M.Z.); katarzyna.osinska@us.edu.pl (K.O.); 2 Department of Physics, Faculty of Science, University of Hradec Králové, Rokitanského 62, 500 03 Hradec Králové, Czech Republic

**Keywords:** ferroelectrics, dielectric properties, PZT-type material, admixtures, sintering

## Abstract

In this work, a multicomponent PZT-type material doped with manganese Mn, antimony Sb, samarium Sm, and tungsten W was fabricated using classical powder technology. Sintering of the ceramic samples was performed by the free sintering method (pressureless sintering). The influence of samarium on the properties of PZT was analyzed using a variable amount of samarium Sm^3+^ (from 0.8 to 1.2 wt.%) and tungsten W^6+^ (from 1.4 to 1.2 wt.%) admixture compared to the Pb(Zr_0.49_Ti_0.51_)_0.963_Mn_0.021_Sb_0.016_O_3_ + W^6+^1.8 wt.% reference composition. XRD studies have shown that PZT-type ceramic samples have a tetragonal structure with a point group of P4*mm*. Field emission scanning electron micrographs (FE-SEMs) showed fine and properly crystallized grains with an average grain size of 5.65–7.70 μm and clearly visible grain boundaries. The polarization–electric field (*P-E*) hysteresis measurement confirmed the ferroelectric nature of the ceramic materials with high *P*_m_ maximum polarization values (from 12.38 to 16.46 μC/cm^2^). Dielectric studies of PZT-type materials have revealed high permittivity values (from 1025 to 1365 at room temperature (RT) and from 18,468 to 25,390 at phase transition temperature *T*_m_) with simultaneously low tan*δ* dielectric loss factor values (from 0.004 to 0.011 at RT) and low DC electrical conductivity, which are important parameters for microelectronic applications. The most homogeneous structure and the most favorable set of utility parameters are represented by the composition with an equal content of Sm and W admixtures, i.e., for 1.2 wt.%.

## 1. Introduction

For many decades, in the field of microelectronics and micromechatronics, ferroelectric ceramic materials obtained based on PbZr_1−*x*_Ti*_x_*O_3_ (PZT) solid solution have been the main focus of interest due to their functional dielectric, ferroelectric, and piezoelectric properties [1,2,3,4]. Their versatile application possibilities depend on the selection of the percentage of zirconium/titanium (Zr/Ti) in the PZT solid solution and the use of appropriate doping and synthesis and sintering technology with optimal process parameters [1,5,6,7]. The greater application possibilities for wide use are shown by multicomponent PZT materials doped with various modifiers and multicomponent solid solutions obtained based on PZT [3,8,9,10,11]. The PZT material has a perovskite-type structure (ABO_3_), in which Ti^4+^ and Zr^4+^ cations occupy the B positions randomly, while lead cations Pb^2+^ occupy the A positions of the compound. The first phase diagram of PZT was presented by Jaffe et al. [12], which shows the change in the crystal structure and related physical properties with the change in the Zr/Ti ratio. In the range from 100/0 to 96/04 (Zr/Ti), PZT is an antiferroelectric (orthorhombic symmetry); in the range from 96/04 to 53/47, the material is a ferroelectric (rhombohedral structure); and in the range from 53/47 to 0/100, PZT is a ferroelectric (tetragonal structure). Between the tetragonal and rhombohedral phases, there is a morphotropic boundary (both phases coexist) [13,14,15,16,17]. PZT compositions close to the morphotropic boundary exhibit maximum piezoelectric activity and extreme functional parameters [18,19,20].

Making a change in the Zr/Ti ratio in PZT materials can be obtained for a wide range of applications in many areas of piezoelectronics, e.g., broadband electrical filters, accelerometers and actuators, ultrasound generators, pressure sensors, piezoelectric transformers, SAW devices, and energy storage devices [19,21,22,23,24,25,26,27,28,29]. Due to high sintering temperatures, the chemical composition of PZT-type solid solutions often deviates from stoichiometry and typically contains free oxygen vacancies and/or lead vacancies. The electrical conductivity of ferroelectric materials, i.e., free charge carriers, depends on their concentration ratio. If cationic vacancies predominate, the material exhibits hole conductivity (p-type), while if anion vacancies predominate in the crystal lattice, the material exhibits electronic conductivity (n-type). Pure PZT material shows p-type conductivity, which is associated with increased oxidation, resulting in an excess of lead (cationic) vacancies over oxygen (anionic) vacancies [12,22]. One of the most effective ways to improve the functional parameters of PZT-type perovskite materials is to modify their base composition with dopants of a higher or lower oxidation state [30,31,32]. On this basis, many methods of heterovalent doping are distinguished, including soft doping, hard doping, middle hard doping, and complex doping. Soft dopants are cations with a higher oxidation state than the base composition cations (Pb^2+^ or Zr^4+^, Ti^4+^) with similar radii [33,34]. The consequence of soft doping is the formation of lead vacancies in the PZT crystal lattice. Soft doping increases the ferroelectric softness of PZT, i.e., the permittivity (*ε*), electrical specific resistance (*ρ*_s_), electromechanical coupling coefficient (*k*_p_), elastic susceptibility, and internal friction ($Q_{m}^{-1}$) increase, while the coercive field (*E*_c_), electrical quality factor (*Q*_e_), and mechanical quality factor (*Q*_m_) decrease [5,34]. Hard admixtures are cations with a lower oxidation state than the base cations in the PZT unit cell; therefore, in order to maintain electrical neutrality, oxygen vacancies are formed in the crystal lattice [12,22]. Hard dopants increase the ferroelectric hardness, i.e., they reduce the value of parameters *ε*, tan*δ*, *k*_p_, *ρ*_s,_ and $Q_{m}^{-1}$, whereas *E*_c_, *Q*_m_, and *Q*_e_ increase, along with the temporary stability of functional parameter PZT materials. Hard dopant cations inhibit the growth of grains in the sintering process due to their low solubility in the crystal lattice of the PZT solid solution. Some hard dopant cations that do not dissolve in the crystal lattice accumulate on the grain boundaries, inhibiting their growth. The presence of the precipitated phase on the grain boundaries strengthens the bonding of neighboring grains, which increases the values of elastic constants and mechanical strength. Complex dopants are systems of two or more elements introduced into the basic PZT composition [25,32,35,36]. This combination gives better results than doping in the form of a single modifier, integrating their individual features, and rare earth elements are often used in this type of doping [37,38]. Thus, modifying the chemical composition of a PZT-type material with oxides of various metals can cause, among others, changes in the crystal structure (i.e., changes in the parameters of the crystal lattice, deformation of the elementary cell, widening and shifting of the morphotropic boundary, change in the concentration of vacancies), changes in the microstructure (i.e., inhibition or intensification of grain growth, increase in the concentration of the intergranular phase), and changes in electrical conductivity (i.e., increase or decrease in electrical conductivity, change in the type of electrical conductivity) [12,22].

In this work, complex doping of PZT was used, and the influence of the samarium Sm dopant on the properties of PZT was investigated. Three compositions of multicomponent PZT ceramics doped with manganese Mn, antimony Sb, samarium Sm, and tungsten W (for different Sm/W contents), and a reference PZT composition (without samarium dopant), were obtained by the pressureless sintering method. In the experiment, by carrying out complex doping in PZT, we aimed to compensate for the formation of lead and oxygen vacancies and, by combining the advantages of hard and soft doping, obtain a beneficial effect on the electrophysical properties of the PZT-type material. The acceptor dopant Sb^3+^ is designed to increase spontaneous polarization (*P*_s_) [39]. In contrast, the donor dopant W^6+^ increases the residual polarization (*P*_r_) and the donor dopant Sm^3+^ increases the permittivity values (*ε*) and reduces the coercive field (*E*_c_) [40]. The isovalent dopant Mn^4+^ is designed to increase the grain homogeneity in the PZT-type ceramic microstructure [38]. Comprehensive X-ray, DC electrical conductivity, and microstructural, dielectric, and ferroelectric property studies of the obtained compositions were carried out. A positive effect of samarium doping on the base composition on the functional parameters of the PZT-type material was demonstrated.

## 2. Materials and Methods

### 2.1. Material and Technological Process

This paper presents the technological process of carrying out, and the results of, electrophysical tests four multicomponent PZT compositions, i.e., (i) Pb(Zr_0.49_Ti_0.51_)_0.963_Mn_0.021_Sb_0.016_O_3_ + W^6+^1.8 wt.% (P) reference composition and three compositions doped with samarium (in which double doping was used with a variable amount of Sm/W), i.e., (ii) Pb(Zr_0.49_Ti_0.51_)_0.963_Mn_0.021_Sb_0.016_O_3_ + Sm^3+^0.8 wt.% + W^6+^1.4 wt.% (P-Sm8), (iii) Pb(Zr_0.49_Ti_0.51_)_0.963_Mn_0.021_Sb_0.016_O_3_ + Sm^3+^1.0 wt.% + W^6+^1.3 wt.% (P-Sm10), and (iv) Pb(Zr_0.49_Ti_0.51_)_0.963_Mn_0.021_Sb_0.016_O_3_ + Sm^3+^1.2 wt.% + W^6+^1.2 wt.% (P-Sm12). The material for the tests was obtained using classic powder technology, and the same technological process conditions were used for all compositions. The starting reagents were simple oxides, i.e., ZrO_2_ and TiO_2_ (99.99% purity, Merck, Darmstadt, Germany), PbO (99.99% purity, POCH, Gliwice, Poland), MnO_2_ and Sb_2_O_3_ (99.995% purity, Aldrich, Saint Louis, MI, USA), and WO_3_ (99.9% purity, Fluka, Bucharest, Romania). In the case of the (ii)–(iv) compositions, an appropriate amount of samarium was added Sm_2_O_3_ (99.5% purity, Aldrich, Saint Louis, MI, USA). Component powders weighed in stoichiometric amounts were mixed in a planetary ball mill (Fritsch Pulwerisette-6, Idar-Oberstein, Germany) for 24 h. For all compositions, wet mixing was used in ethyl alcohol using zirconium balls. After mixing, the powders were dried and then synthesized by calcination at 850 °C for 4 h with a heating rate of 150 °C/h. After calcination, the powders were mixed and homogenized, and then disk-shaped ceramic compacts were made on a hydraulic press (with dimensions *ϕ* = 10 mm and thickness *d* = 3 mm). Sintering of the PZT-type ceramic samples was carried out in a muffle furnace under the conditions 1150 °C for 2 h and a heating rate of 150 °C/h. After sintering, the surfaces of the ceramic samples were grinded and polished, and next, they were annealed to remove mechanical stress (700 °C/15 min.). For electrical tests, silver electrodes were applied to both surfaces of the samples (using the silver paste burning method).

### 2.2. Characterization

The X-ray diffraction measurements were taken with a Malvern Panalytical Empyrean diffractometer using nickel-filtered CuK_α1,2_ radiation (*λ* = 1.5406 Å) and equipped with a PIXcell^3D^ ultra-fast solid-state hybrid detector (Malvern Instruments, Malvern, UK). The measurements were taken at room temperature (RT) in a reflection mode, in the Bragg–Brentano geometry (*θ*–*θ* scan technique), within the 2*θ* range of 10–120°. The Pawley refinement was performed using the FullProf program suite (Version 7.95-Jan2023-ILL JRC) [41]. The microstructure surface images and chemical composition analysis (Energy Dispersive Spectroscopy, EDS) were performed on Jeol field emission scanning electron microscopy (FE-SEM, JSM-7100F TTL LV, Tokyo, Japan) with the EDS analysis system. The relative density of the ceramic samples was specified using the Archimedes method. The dielectric measurements were made on the QuadTech 1920 Precision LCR meter (Maynard, MA, USA) in the temperature range from 20 to 450 °C. The DC electric conductivity measurements were taken on a precise electrometer (Keithley 6517B, Cleveland, OH, USA) in the temperature range from 20 to 450 °C. The tests of the *P-E* ferroelectric hysteresis loops were made at RT on the Sawyer–Tower circuit and a high-voltage amplifier (Matsusada Inc. HEOPS-5B6 Precision, Kusatsu, Japan) [42]. The measurement data were obtained by digital card (National Instruments Corporation, Austin, TX, USA) in cooperation with a computer program created in the LabView environment.

## 3. Results and Discussion

### 3.1. Structural Measurement

**Figure 1:**
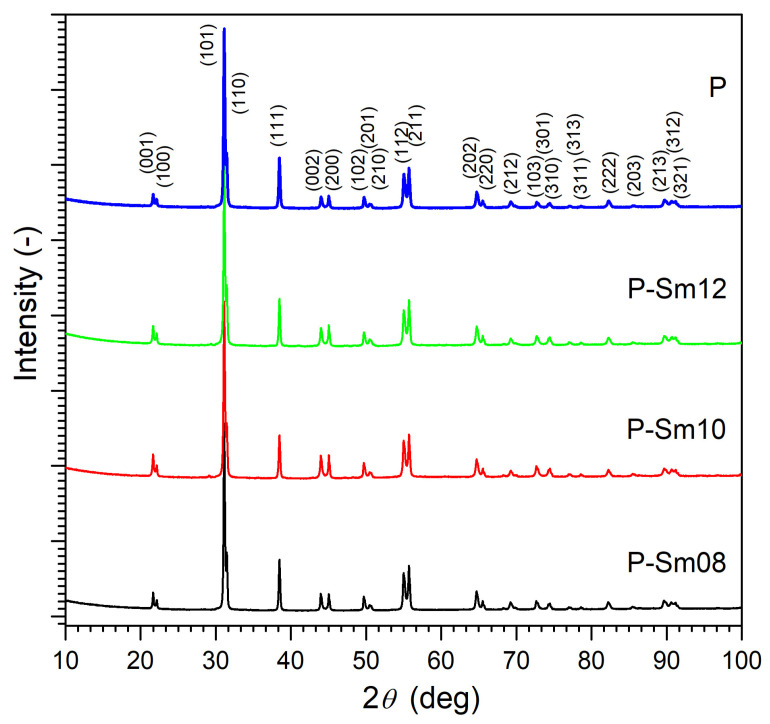
X-ray pattern of the PZT-type samples: P, P-Sm8, P-Sm10, and P-Sm12.

**Figure 2:**
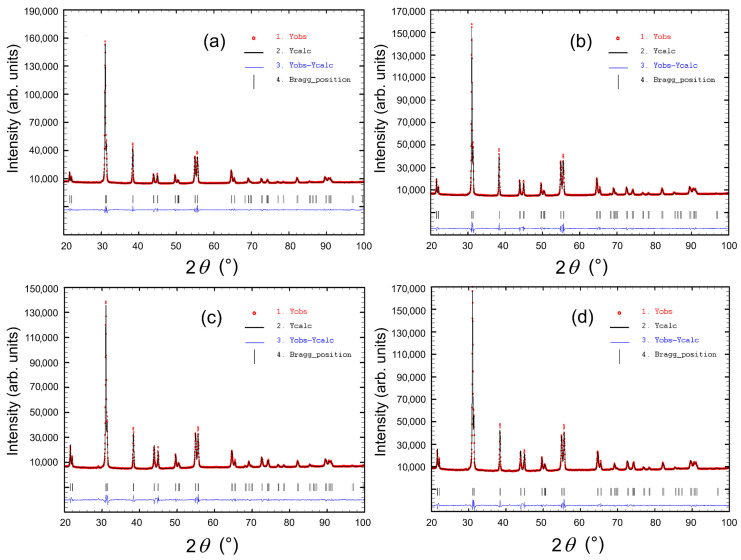
Pawley’s refinement of the PZT-type samples: (**a**) P, (**b**) P-Sm8, (**c**) P-Sm10, and (**d**) P-Sm12.

Figure 1 shows the X-ray diffraction patterns of the PZT-type materials tested at room temperature. The main diffraction lines were identified as a tetragonal perovskite structure with space group P4*mm* and were matched to pattern JCPDS #04-006-3340. The XRD measurements also confirmed the occurrence of a single phase without the foreign phases, e.g., the undesirable pyrochlore phase. Pawley’s method was used to calculate the unit cells for the analyzed PZT-type ceramic samples (Figure 2). Based on the obtained results, for compositions with the samarium admixture, a slight increase in the volume of the unit cell was observed (Table 1).

### 3.2. Microstructure Measurement

**Figure 3:**
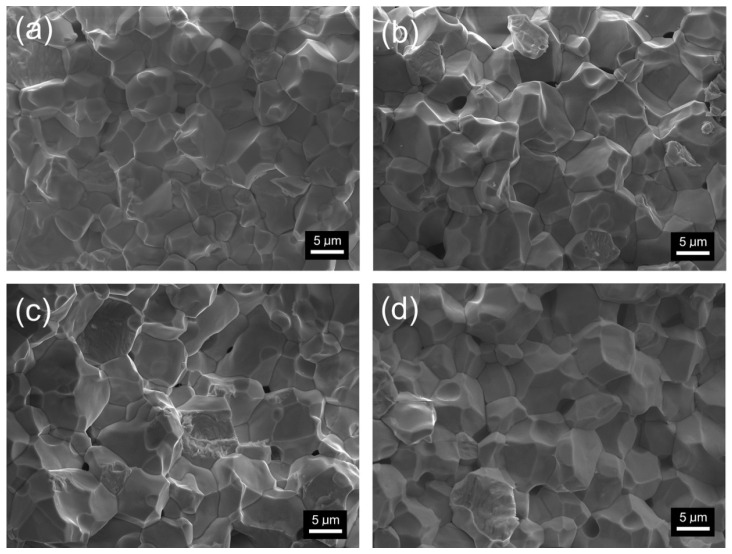
FE-SEM images of the microstructure of the cross-section of the PZT-type sample surfaces: (**a**) P, (**b**) P-Sm8, (**c**) P-Sm10, and (**d**) P-Sm12.

**Figure 4:**
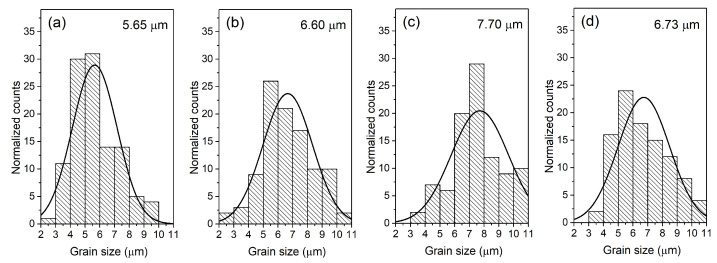
Grain size distribution for the PZT-type sample: (**a**) P, (**b**) P-Sm8, (**c**) P-Sm10, and (**d**) P-Sm12.

Figure 3 shows FE-SEM images of the microstructure fracture of PZT-type solid solution samples. Densely packed and properly crystallized grains characterize the microstructure of the cross-section of the samples. The boundaries of the grains of the microstructure are perfectly visible, and when breaking samples, the inter-grain cracking mechanism dominates. This confirms the high mechanical strength of the interior of the grains of the PZT ceramic samples. The strongly solidified and durable material of the interior of the grain confirms the correctly selected sintering conditions in the technological process of obtaining the ceramic material. FE-SEM microstructural studies also showed that the presence of samarium dopant in the PZT composition promotes grain growth but does not negatively affect the homogeneity of the microstructure grains (Figure 4). The smallest average grain size is shown by the P composition (without samarium dopant) *r*_av_ = 5.65 μm, while for the samarium-doped compositions, *r*_av_ is 6.60, 7.70, and 6.73 μm for P-Sm8, P-Sm10, and P-Sm12, respectively.

The observed increase in grain size upon Sm addition is a microstructural effect that does not necessarily require significant changes in the crystallographic unit cell volume (Table 1). The slight variations in lattice volume between the reference composition and additionally doped samples are consistent with the substitutional incorporation of Sm ions into the crystal structure (with low amounts) in the place of cations with similar ionic radii. While lattice strain and defect-related effects could influence unit cell parameters, our analysis of Pawley’s method did not indicate any need to invoke such contributions to explain the data. Therefore, we attribute the observed grain growth primarily to changes in sintering behavior and grain boundary mobility induced by Sm doping rather than structural distortions.

**Figure 5:**
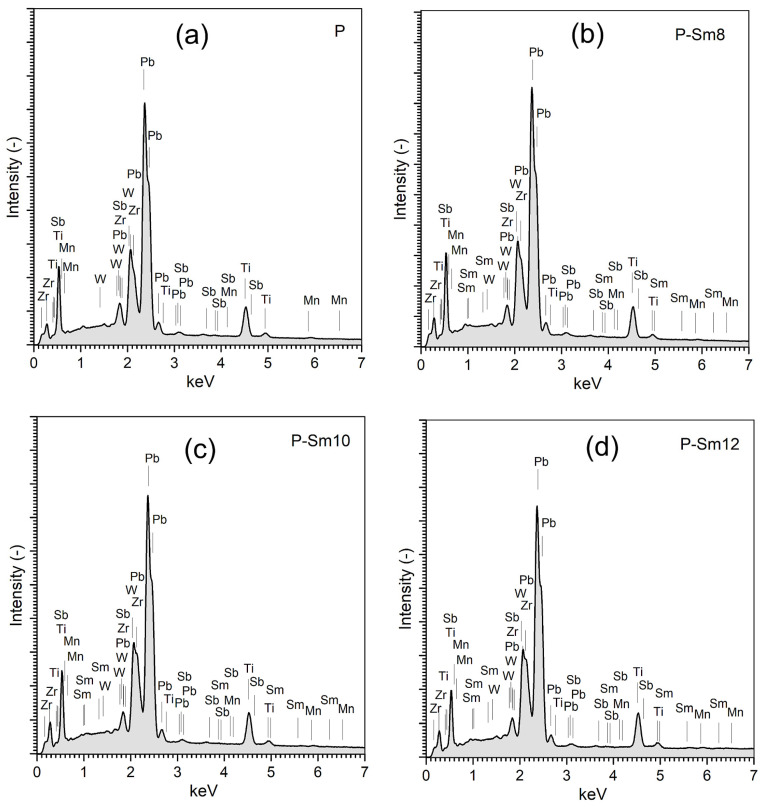
The EDS analysis of the element distribution for the multicomponent PZT-type samples: (**a**) P, (**b**) P-Sm8, (**c**) P-Sm10, and (**d**) P-Sm12.

The surface energy dispersive spectrometry analysis (EDS) of the PZT-type ceramic samples is depicted in Figure 5. EDS analysis was the average results from five randomly selected microstructure areas of the ceramic samples. The EDS study confirmed the assumed chemical composition of the PZT-type materials without foreign elements. The experimental results of the percentage of elements in the PZT-type materials are summarized in Table 2.

PZT ceramic materials exhibit high relative densities (Table 3). The relative density measurement using the Archimedes method showed that the reference P composition has the highest density. At the same time, additional doping with samarium causes a decrease in the density of the samples. This is because ceramics with P composition exhibit a fine-grained structure with firmly packed grains. In compositions with alternating the Sm/W content, there is an increase in grain size and a greater tendency to form internal pores that reduce the density of the material.

### 3.3. Dielectric Tests

**Figure 6:**
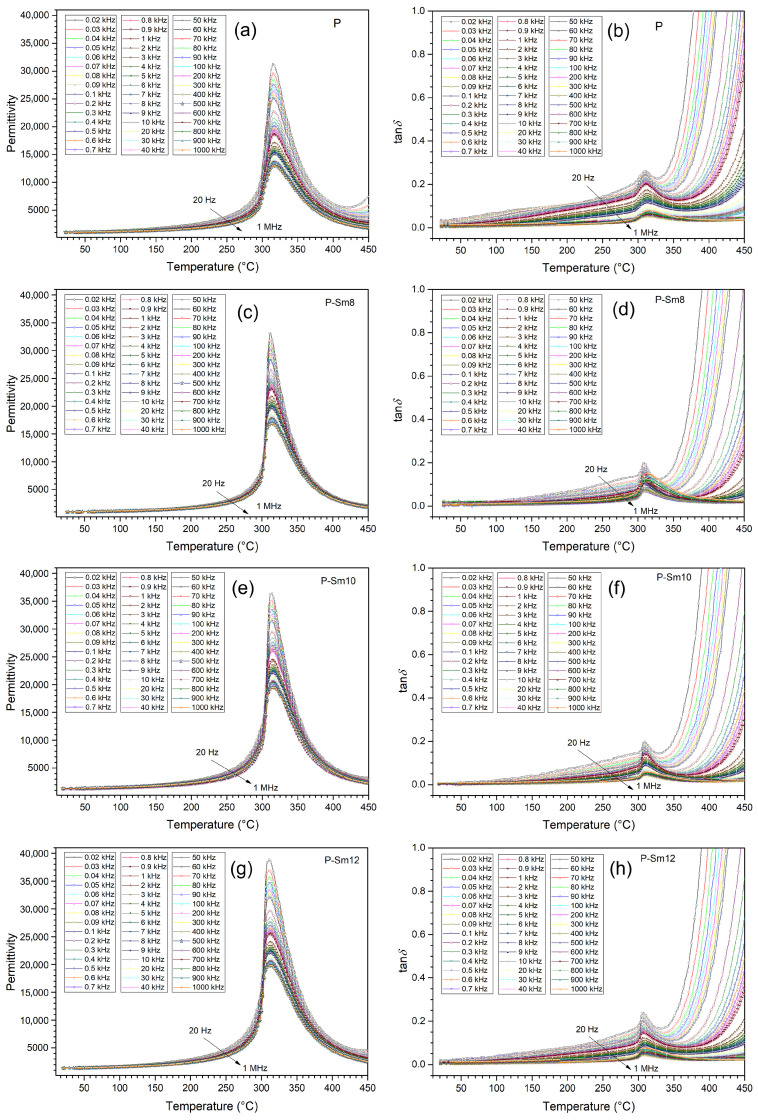
Temperature dependencies of the permittivity (**a**,**c**,**e**,**g**) and dielectric loss factor tan*δ* (**b**,**d**,**f**,**h**) of the PZT-type ceramic samples: (**a**,**b**) P, (**c**,**d**) P-Sm8, (**e**,**f**) P-Sm10, and (**g**,**h**) P-Sm12.

Measurements of the dielectric properties of PZT ceramic materials performed in the temperature range from RT to 450 °C showed high permittivity values with a clearly defined phase transition point (Figure 6). At room temperature, the permittivity *ε* for the P material is 1025, while for the compositions with samarium doping, the permittivity values are higher, i.e., 1040, 1267, and 1365, for P-Sm8, P-Sm10, and P-Sm12, respectively. With increasing temperature, the permittivity values systematically increase to reach a maximum at the phase transition temperature (*T*_m_). At *T*_m_, the doped PZT compositions also have high maximum permittivity values and are 23,987, 24,480, and 25,390, for P-Sm8, P-Sm10, and P-Sm12, respectively (Table 3). For comparison, at *T*_m_, the *ε*_m_ value of the P material is 18,468. In ferroelectric materials, there is also a phenomenon of a decrease in the permittivity value with increasing frequency, which indicates a low-frequency dispersion phenomenon observed in the temperature permittivity graph. High permittivity values at low frequencies are interpreted by the polarization of the space charge at the grain boundaries, which results in the formation of a potential barrier. The accumulation of space charge at the grain boundary increases the dielectric constant of ceramic materials, while the occurring dielectric dispersion results from the increased effect of the grain boundary interaction compared to the much weaker effect of the grain interaction [43]. At higher frequencies, the electric dipole (space charge) is not reorienting itself enough to follow the applied external field, which results in a decrease in permittivity with increasing frequency [44].

Ceramic samples with samarium doping are characterized by a sharper phase transition, which indicates an increase in the homogeneity of the structure and order in the structure of the compound compared to the P material (without samarium doping). In undoped PZT ceramics, the increased phase transition blurring is accompanied by a lower decrease in the permittivity value with increasing frequency. At the phase transition temperature *T*_m_, the permittivity decrease (in the frequency range from 20 Hz to 1 MHz) is 41%. The PZT compositions doped with samarium are characterized by a greater difference in the permittivity values at low and high frequencies. At the phase change temperature, the permittivity decrease is 51% for P-Sm8, 53% for P-Sm10, and 51% for P-Sm12. In conclusion, doping PZT with an additional rare earth admixture of samarium positively affects dielectric properties, including an increase in permittivity, “sharpening” the phase transition, and reducing dielectric loss. This indicates an increase in the orderliness of the crystal structure and a greater homogeneity of the distribution of admixtures in the crystal lattice. The improvement in the dielectric properties can be attributed to the introduction of Sm^3+^ donor admixture, which replaces A-site ions and creates additional cationic vacancies in the ceramic materials. The existence of these vacancies makes it easier for the domain walls to move under the action of an electric field. The increase in domain wall mobility means that the material’s polarization is more likely to change under the influence of an external electric field, leading to an increase in the permittivity [45]. The permittivity of the PZT-type ceramics also depends on the microstructure (proportional to the average value of grain size [46]). Higher permittivity values are usually found in ceramic materials with larger grains, while a fine-grained structure causes a decrease in permittivity [41]. This regularity in the analyzed ceramic samples was confirmed by the FESEM microstructural studies presented above. The observed grain growth is primarily attributed to changes in grain boundary mobility induced by Sm doping.

Dielectric property tests confirmed low dielectric loss factor values (tan*δ*) for the analyzed PZT compositions. At RT, the tan*δ* value for the P material is 0.011, while for compositions with samarium admixture, the dielectric loss factor values are much lower and are 0.004, 0.005, and 0.005 for P-Sm8, P-Sm10, and P-Sm12, respectively. At lower frequencies, the dielectric loss of ferroelectric materials is higher than for higher frequencies. In the tan*δ*(*T*) graphs, with increasing temperature, a progressive increase in the value of the dielectric loss factor is observed, with a clear local maximum tan*δ* just before the ferroelectric–paraelectric phase transition. The occurring local maximum tan*δ* is a characteristic feature of PZT ceramic materials [47]. Above 400 °C, there is a rapid increase in dielectric tangent loss, which is associated with the increase in electrical conductivity of ceramic materials at high temperatures.

**Figure 7:**
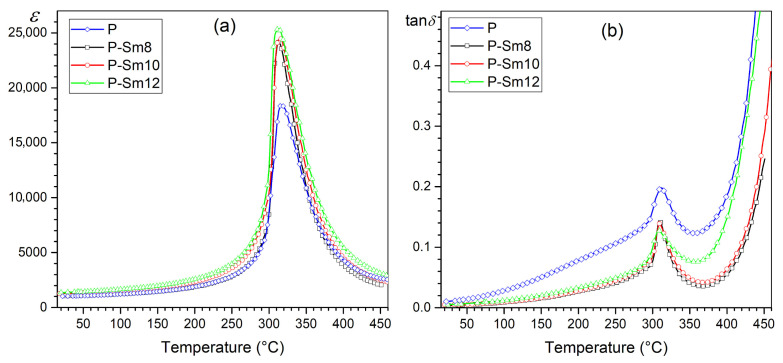
The comparison of the permittivity (**a**) and dielectric loss factor (**b**) for the PZT-type ceramic samples measured at 1 kHz.

Figure 7 presents a comparison of the temperature courses of permittivity *ε* and tan*δ* of the analyzed compositions for the frequency of 1 kHz. The comparison well illustrates the beneficial effect of the samarium dopant introduced into PZT on both dielectric parameters of ceramic materials. The samarium dopant effectively increases the permittivity values by narrowing the phase change region and at the same time reduces the dielectric loss factor values of PZT ceramic materials. Partial lead replacement by samarium of the perovskite structure can alter the crystal structure, introduce defects, and impact charge carriers within the material. These changes influence polarization dynamics and relaxation processes, contributing to changes in dielectric loss. The Sm admixture in the A-site may create more defect sites or alter existing ones, affecting charge carrier mobility and dielectric loss behavior. When doping improves the crystal structure or neutralizes existing defects, the dielectric loss might decrease as the material becomes more ordered and polarization dynamics become more efficient [48]. Sm^3+^ ions replacing Pb^2+^ ions in PZT generate vacancies in the A-site, which neutralizes the charge imbalance.

### 3.4. DC Conductivity Test

**Figure 8:**
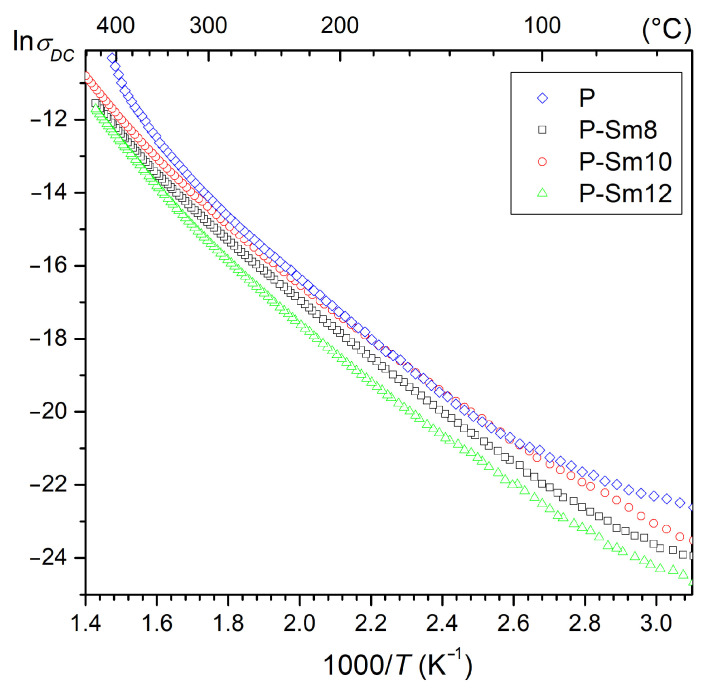
The ln*σ*_DC_ (1000/*T*) relationship for the PZT-type ceramic materials.

The PZT-type ceramic samples doped by Sm have high values of *ρ*_DC_ resistivity, e.g., at 50 °C, 1.75 × 10^10^ Ωm (for P-Sm8), 2.64 × 10^10^ Ωm (for P-Sm10), and 5.45 × 10^10^ Ωm (for P-Sm12), and are higher than for the undoped composition P (4.91 × 10^9^ Ωm). The increase in temperature systematically increases the electrical conductivity of ceramic materials. At higher temperatures, i.e., above 300 °C, the increase in conductivity is greater, where a change in the slope of the curves in the ln*σ*_DC_ (1000/*T*) graph is observed. Based on the slopes of the fitted curves to the experimental plots, the activation energy was calculated according to the Arrhenius law (1) at lower (<300 °C) and higher (>300 °C) temperatures.

(1) $$\sigma_{DC}=\sigma_{0}exp\left(\frac{E_{a}}{k_{B}T}\right),$$

where *σ*_0_—pre-exponential factor, *k*_B_—Boltzmann constant, *E*_a_—activation energy, and *T*—absolute temperature [49]. The calculated values of the activation energy are listed in Table 3. At a lower temperature range (<300 °C), the activation energies *E*_a_ of the ceramic samples are 0.71 eV (for P), 0.65 eV (for the P-Sm8), 0.62 (for the P-Sm10), and 0.65 eV (for the P-Sm12). At higher temperatures (>300 °C), the activation energies *E*_a_ are higher, i.e., 1.13 eV (for P), 0.93 eV (for the P-Sm8), 0.91 eV (for the P-Sm10), and 1.01 eV (for the P-Sm12). Above 350 °C, the increase in conductivity is greater for sample P (Figure 8).

Generally, in most perovskite materials, conductivity is connected mainly with dipolar defect effects and oxygen and lead vacancies [46,50]. Conduction in lower-temperature areas is mostly related to ionization processes, i.e., electrons or holes, which are the dominant charge carriers. In higher-temperature areas, there is an intensification of the activation of external defects, and their mobility is the dominant factor influencing electrical conductivity. A further increase in temperature causes an increase in the concentration and movement of internal defects, which plays a dominant role in the conduction process of ceramic materials at very high temperatures [51,52,53]. In the present study of the PZT-type samples, the calculated *E*_a_ activation energy ranges from 0.62 eV to 1.13 eV, which indicates that the dominant conductivity factor in the ceramic materials is the presence of oxygen vacancies, i.e., doubly ionized oxygen vacancies [50,53].

### 3.5. Ferroelectric Test

**Figure 9:**
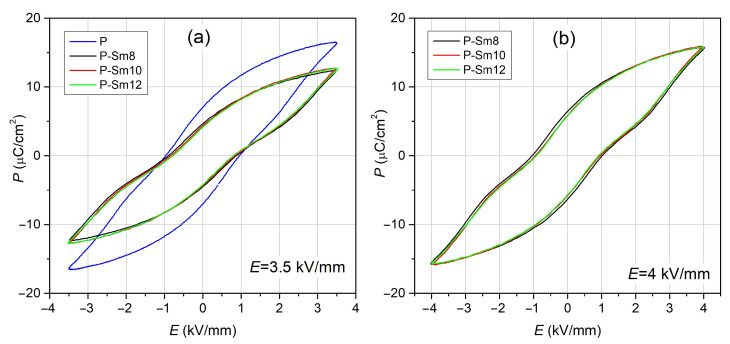
The *P-E* ferroelectric hysteresis loops for the PZT-type ceramic materials: (**a**) test for 3.5 kV/mm and (**b**) test for 4 kV/mm.

Figure 9 depicts the *P-E* hysteresis loops of the PZT-type ceramic samples at RT. The *P-E* research showed the resultant ferroelectric properties of PZT ceramic samples, i.e., intermediate properties between ferro-hard and ferro-soft materials, and a narrowing of the loop occurs at polarization *P* = 0. For the reference composition P at RT, the residual polarization *P*_r_, the maximum polarization *P*_m_, and the coercive field *E*_c_ values measured at 3.5 kV/mm are 7.27 μC/cm^2^, 16.45 μC/cm^2^, and 0.97 kV/mm, respectively (Figure 9a, Table 3). In the case of samarium-doped compositions, the polarization values are lower and differ only slightly; *P*_r_ in the range of 4.24–4.65 μC/cm^2^ and *P*_m_ in the range of 12.38–12.72 μC/cm^2^. With the increase in the samarium dopant amount, the coercive field *E*_c_ of PZT ceramic materials decreases. *E*_c_ is 0.89 kV/mm for P-Sm8, 0.84 kV/mm for P-Sm10, and 0.78 kV/mm for P-Sm12. The lower coercive field of the doped compositions allows for easier repolarization of the loop, and their lower conductivity allows for the application of a stronger external field *E* and for higher polarization values to be obtained (Figure 9b, Table 3).

The sintering method, thermal treatment, mechanical stresses, and charged defects are factors that may affect the shape of the ferroelectric loop and coercive field (*E*_c_) and remnant polarization (*P*_r_) values [54,55]. Doping with ions of different valence states causes several changes that influence the electrical properties of PZT ceramic materials. Mostly, doping with higher-valence ions will lead to the generation of lead (Pb) vacancies, which is conducive to the deflection of the domain in the electric field, resulting in *P*_r_ increasing and *E*_c_ decreasing. In contrast, replacing the high-valence ions with low-valence ions will generate oxygen vacancies and the pinning effect. This will increase *E*_c_ and decrease *P*_r_ values in PZT-type ceramic materials [32,56,57]. The use of complex doping in PZT resulted in the combination of the above effects. The P composition (without Sm dopant) shows high *P*_r_ values and higher *E*_c_, while the Sm/W-doped compositions have lower *P*_r_ values and lower coercive field *E*_c_. Comparing the SEM microstructural images of PZT ceramic materials, it can be concluded that the higher coercive field *E*_c_ is related to the finer grain size in the microstructure of the P composition, which is attributed to the difficulty in reversing the polarization of domains in the fine-grained microstructure. The increase in the average grain size in the microstructure facilitates the reversal of domain polarization, increasing the coercive field value. As the grain size reduces in the microstructure, the defects and grain boundaries possess pinning effects on the domains, causing an enlargement of *E*_c_ values [58].

## 4. Conclusions

This work obtained and tested a multicomponent PZT-type material doped with manganese Mn, antimony Sb, samarium Sm, and tungsten W with variable Sm/W content (Sm^3+^ from 0.8 to 1.2 wt.% and W^6+^ from 1.4 to 1.2 wt.%). The reference composition was as follows: Pb(Zr_0.49_Ti_0.51_)_0.963_Mn_0.021_Sb_0.016_O_3_ + W^6+^1.8 wt.%. This experience has confirmed that the appropriate selection of admixtures (isovalent, acceptor, and donor) and correctly conducted technological process (with optimal sintering parameters) allows for obtaining high densities of PZT ceramic samples using the classic sintering method (pressureless sintering). All the above factors positively influence the electrophysical parameters of ceramic materials. XRD studies have shown that the multicomponent PZT-type ceramic samples have a tetragonal structure with a point group of P4*mm*. Properly crystallized grains with sharp and clearly visible grain boundaries characterize the microstructure of ceramic samples. Dielectric studies of PZT-type materials have revealed high permittivity values (for 1 kHz at RT they range from 1025 to 1365 and at *T*_m_ temperature phase transition they range from 18,468 to 25,390). Also, the PZT-type materials show low dielectric loss factor values (tan*δ*) at RT (0.004–0.011). The Sm samarium dopant introduced into the PZT compound benefits the dielectric properties of the PZT-type solid solution, i.e., an increase in the permittivity value and a significant reduction in dielectric loss and DC electrical conductivity. The ferroelectric–paraelectric phase transition occurs in a narrower temperature range, which indicates a high degree of structural order in the PZT-type compound. The samarium dopant also has a beneficial effect on the ferroelectric properties, maintaining high polarization values. Due to the higher resistivity at room temperature compared to the PZT sample without samarium dopant, it is possible to use higher polarization fields. The studies have shown that doping the PZT-type solid solution with samarium is suitable for obtaining favorable properties for use in micromechatronic applications, such as precision actuators and micro-shifters.

## Figures and Tables

**Table 1:** Summary of refined Pawley’s method unit cell parameters for the PZT-type ceramic samples.

Parameter	P	P-Sm8	P-Sm10	P-Sm12
Lattice parameter *a* (Å)	4.0336(1)	4.0350(1)	4.0334(1)	4.0332(1)
Lattice parameter *c* (Å)	4.1110(1)	4.1244(1)	4.1237(1)	4.1219(1)
Lattice volume *V* (Å^3^)	67.03(1)	67.15(1)	67.09(1)	67.05(1)
*R_p_*	17.3	13.5	14.1	14.1
*R_wp_*	12.3	11.6	12.5	12.0
*Chi* ^2^	12.2	11.2	12.5	13.8

The reduced *Chi*^2^ is defined in the FullProf software as $\chi^{2}={\left(\frac{R_{wp}}{R_{exp}}\right)}^{2}$, where *R*_wp_ is the weighted profile *R*-factor and *R*_exp_ is the expected *R*-factor.

**Table 2:** Experimental percentages of the individual components of the PZT-type ceramic samples.

Oxide	P	P-Sm8	P-Sm10	P-Sm12
PbO	74.41	73.59	73.35	72.47
ZrO_2_	13.55	13.40	13.08	12.66
TiO_2_	10.39	11.08	11.25	12.34
MnO_2_	0.33	0.47	0.55	0.60
Sb_2_O_3_	0.70	0.51	0.52	0.56
Sm_2_O_3_	-	0.45	0.77	1.05
WO_3_	0.62	0.50	0.47	0.33

**Table 3:** Parameters of the PZT-type ceramic samples.

Parameters		P	P-Sm8	P-Sm10	P-Sm12
*ρ* (g/m^3^)		7.33	7.02	7.15	7.22
*ρ*_DC_ (Ωm) at 50 °C		4.91 × 10^9^	1.75 × 10^10^	2.64 × 10^10^	5.45 × 10^10^
*ε* at RT		1025	1040	1267	1365
*T*_m_ (°C)		317	313	314	313
*ε_m_*		18,468	23,987	24,480	25,390
tan*δ* at RT		0.011	0.004	0.005	0.005
tan*δ* at *T*_m_		0.188	0.132	0.138	0.121
*P*_r_ (μC/cm^2^)	for 4 kV/mm	-	6.52	5.85	5.85
	for 3.5 kV/mm	7.27	4.65	4.42	4.24
*P*_m_ (μC/cm^2^)	for 4 kV/mm	-	15.8	15.9	15.9
	for 3.5 kV/mm	16.45	12.38	12.70	12.72
*E*_c_ (kV/mm)	for 4 kV/mm	-	1.03	0.97	0.92
	for 3.5 kV/mm	0.97	0.89	0.84	0.78
*E*_a_ < 300 °C (eV)		0.71	0.65	0.62	0.65
*E*_a_ > 300 °C (eV)		1.13	0.93	0.91	1.01

## Data Availability

The original contributions presented in this study are included in the article. Further inquiries can be directed to the corresponding authors.
